# Antimicrobial drug-related problems in a neonatal intensive care unit

**DOI:** 10.5935/0103-507X.20170040

**Published:** 2017

**Authors:** Bruna Meirelly Nunes, Tatiana Costa Xavier, Rand Randall Martins

**Affiliations:** 1 Multiprofessional Health Residence, Maternidade Escola Januário Cicco, Universidade Federal do Rio Grande do Norte - Natal (RN), Brazil.; 2 Department of Pharmacy, Universidade Federal do Rio Grande do Norte - Natal (RN), Brazil.

**Keywords:** Medication errors, Anti-infective agents, Intensive care units, neonatal, Aminoglycosides, Infant, newborn

## Abstract

**Objective:**

The goal was to determine the main drug-related problems in neonates who were using antimicrobials.

**Method:**

This was an observational, prospective and longitudinal study. Drug-related problems were classified according to version 6.2 of the Pharmaceutical Care Network Europe Foundation classification. A descriptive analysis was performed, in which the clinical and therapeutic variables were presented as absolute and relative frequencies or as the mean and standard deviation, as appropriate.

**Results:**

In total, 152 neonates with a predominance of males (58.5%), gestational age of 32.7 ± 4.2 weeks and weight of 1,903.1 ± 846.9g were included. The main diagnostic hypothesis of infection was early sepsis (66.5%), and 71.7% of the neonates had some risk factor for infection. Among the neonates, 33.6% had at least one drug-related problem. Of these, 84.8% were related to treatment effectiveness and 15.2% to adverse reactions. The main cause of drug-related problems was the selected dose, particularly for aminoglycosides and cephalosporins.

**Conclusion:**

The use of antimicrobials in the neonatal intensive care is mainly associated with problems related to medication effectiveness, predominantly the prescription of subdoses of antimicrobials, especially aminoglycosides.

## Abstract (pt)

**Objetivo:**

Determinar os principais problemas relacionados a medicamentos em neonatos sob uso de antimicrobianos.

**Métodos:**

Estudo observacional, prospectivo e longitudinal. Os problemas relacionados a medicamentos foram classificados de acordo com a versão 6.2 da *Pharmaceutical Care Network Europe Foundation*. Foi executada análise descritiva, na qual as variáveis clínicas e terapêuticas foram apresentadas por frequências absolutas e relativas, ou por média e desvio padrão, conforme apropriado.

**Resultados:**

Foram incluídos 152 neonatos com predomínio do sexo masculino (58,5%), idade gestacional de 32,7 ± 4,2 semanas e peso de 1.903,1 ± 846,9g. A principal hipótese diagnóstica de infecção foi a sepse precoce (66,5%), detectando-se que 71,7% dos neonatos apresentavam algum fator de risco para infecção. Dentre os neonatos, 33,6% apresentaram pelo menos um problema relacionado a medicamento. Destes, 84,8% estavam relacionados à efetividade do tratamento e 15,2% a reações adversas. A principal causa de problemas relacionados a medicamentos foi a escolha da dose, sobretudo dos aminoglicosídeos e das cefalosporinas.

**Conclusão:**

O uso de antimicrobianos em terapia intensiva neonatal relaciona-se principalmente a problemas relacionados a medicamentos de efetividade, predominando a prescrição de antimicrobianos em subdose, sobretudo os aminoglicosídeos.

## INTRODUCTION

Antimicrobials are widely prescribed in neonatal intensive care units (NICUs).^(1)^ Even neonates who are not afflicted with proven infections often use this type of medication during their hospital stay.^(2)^ However, their use in intensive care is complicated due to the need to select a drug based on its efficacy against the microorganism causing the disease and due to the dosage complexity, which can lead to therapeutic failure, bacterial resistance or toxicity.^(3)^

The main clinical condition that leads to the use of antimicrobials in a NICU is neonatal sepsis, the main cause of morbidity and mortality. To reduce the occurrence of the therapeutic failure associated with the use of these drugs, choosing an adequate antimicrobial regimen is necessary. However, given the difficulties in isolating and identifying the etiological agent, the empirical use of several therapeutic schemes is common.^(4)^ Furthermore, the use of antimicrobials in neonates represents a greater risk from a pharmacokinetic point of view, since the functional immaturity of the organs involved in this process alters the drug metabolism and excretion profile, contributing to an increase in systemic exposure and greater toxicity.^(5)^ The increased risk and therapeutic complexity inherent in the use of antimicrobials in NICUs may be associated with the occurrence of drug-related problems (DRPs).

The Pharmaceutical Care Network Europe defines a DRP as a "drug-related event or circumstance that actually or potentially interferes with desired health outcomes."^(6)^ Studying the occurrence of DRPs with the use of antimicrobials is necessary because of their high frequency.^(7)^ Gentamicin and vancomycin are the antimicrobials most associated with medication errors, and these errors are mainly related to the administration interval.^(8)^

Neonates, in particular, are quite susceptible to DRPs, and this susceptibility may be associated with clinical heterogeneity, such as weight, gestational age and postnatal age - the determinants of dose selection. The explanation for the occurrence of DRPs in this specific group may also be related to the limited number of studies and to the different dosing recommendations found in the available references.^(9)^

The objective of this study was to determine the main DRPs in neonates undergoing antimicrobial therapy in a NICU.

## METHODS

This was an observational, prospective and longitudinal study conducted in the NICU of the *Maternidade Escola Januário Cicco* (MEJC), in the city of Natal, Rio Grande do Norte (RN), Brazil. The MEJC is a reference institution for high-risk pregnancy, gynecological surgery and women's health in the state of RN. The institution currently has 6 adult intensive care unit (ICU) beds and 23 NICU beds.

The convenience sample consisted of newborns treated with antimicrobial therapy from October 2015 to October 2016, totaling 152 neonates. All patients admitted to the NICU during the study period were included in the study, provided they were using at least one antimicrobial agent. Patients whose hospital stay time was less than 24 hours, readmitted patients and those whose data in the pharmacotherapeutic follow-up form were incomplete in terms of the requirements for the study.

The instrument used to collect data was the pharmacotherapeutic follow-up form used by the Clinical Pharmacy Service of the institution to follow up on the neonates hospitalized in the NICU. This instrument includes the biodemographic and pharmacotherapeutic data necessary to carry out the study. The following data were analyzed: sex, gestational age, birth weight, type of delivery, time of ruptured membranes, Apgar score, diagnostic hypothesis, death, blood culture results and presence of maternal risk factors for infection. In addition, the occurrence of DRPs was analyzed, as well as their classifications. The DRPs were classified according to type, causes and performed interventions according to the Pharmaceutical Care Network Europe classification. The adequacy of the dose selected was evaluated using Neofax^®^ 2011^(10)^ and the electronic databases Micromedex^®(11)^ and UptoDate^®(12)^ as sources of information. The DRPs were analyzed in relation to their potential damage but not according to whether the patient was harmed.

Statistical analysis was performed with Stata version 12 (Stata Corporation, College Station, TX, USA). Descriptive analysis was performed, and clinical and therapeutic variables were presented as absolute and relative frequencies or as the mean and standard deviation, as appropriate. The present study was approved by the Research Ethics Committee of the *Hospital Universitário Onofre Lopes* of the *Universidade Federal do Rio Grande do Norte*, under the protocol 580.201/2014 (CAAE: 21718113.3.0000.5292), in accordance with the guidelines of National Health Council Resolution 466/12. All participants gave written informed consent to participate in the study.

## RESULTS

In total, 152 neonates were included in the study; 34 were excluded because they did not use antimicrobials during hospital stay, 26 because of the lack of data needed to perform the study and 7 because they remained hospitalized for less than 24 hours, leading to a total of 67 excluded patients.

The sample consisted mainly of male patients (58.5%), with a mean gestational age of 32.7 ± 4.2 weeks and weight of 1,903.1 ± 846.9g. The patients were hospitalized for an average of 18.1 days. There was a predominance of cesarean delivery (66.5%) with membrane rupture mainly at delivery (72.4%) and Apgar score of 6.5 in the first minute of life and 8.1 in the fifth minute. The main diagnostic hypothesis of infection that was evaluated in the neonates was early sepsis, which corresponded to 66.5% of the total, and 71.7% presented some risk factor for developing infection. Of the blood cultures performed, the result was positive in only 10.5% of the neonates. The characteristics of the sample are described in table 1.

**Table 1:** Sample characteristics

Characteristics	Values
Gestational age (weeks)	32.7 ± 4.2
Male gender	89 (58.5)
Birth weight (g)	1,903.1 ± 846.9
Length of hospital stay (days)	18.1 ± 20.3
Cesarean delivery	101 (66.5)
Time of membrane rupture	
At delivery	110 (72.4)
< 18 hours	24 (15.8)
> 18 hours	18 (11.8)
Apgar 1 minute	6.5 ± 2.3
Apgar 5 minutes	8.1 ± 1.4
Diagnostic hypothesis	
Early sepsis	101 (66.5)
Early sepsis/late sepsis	20 (13.2)
Early sepsis/pneumonia	8 (5.3)
Late sepsis	8 (5.3)
Pneumonia	7 (4.6)
Other	8 (5.3)
Positive blood cultures	16 (10.5)
Mechanical ventilation	94 (61.8)
Maternal risk factor	109 (71.7)
Death	15 (9.9)

Results are expressed as the number (%) or mean ± standard deviation.

For the profile of prescribed drugs (Table 2), 1,149 items were administered, with a predominance of gentamicin (12.1%), aminophylline (7.6%), cefazolin (6.6%), vitamin C (4.6%), amikacin (4.4%) and cefepime (4.4%).

**Table 2:** Most prescribed antimicrobial agents

Characteristics	Values
Drugs prescribed per patient	7.6 ± 5.3
Prescribed drugs	
Gentamicin	139 (12.1)
Aminophylline	87 (7.6)
Cefazolin	76 (6.6)
Vitamin C	53 (4.6)
Cefepime	51 (4.4)
Amikacin	51 (4.4)
Vitamin A + D	49 (4.3)
Fentanyl	47 (4.1)
Omeprazole	46 (4.0)
Dobutamine	44 (3.8)
Vitamin K	40 (3.5)
Others	466 (40.6)
Total	1,149 (100)

Results are expressed as the number (%) or mean ± standard deviation.

Regarding the profile of DRPs, 51 of the 152 patients analyzed had at least one DRP (33.6%). All were classified as potential problems. Of these, 84.8% were related to the effectiveness of treatment, and 15.2% were adverse reactions (Table 3), with the main cause being the dose selected (subdose, overdose and frequency of administration) in 72.1% of the patients; the DRPs were mainly associated with aminoglycosides, followed by cephalosporins (Table 4).

**Table 3:** Drug-related problems and results of interventions

Type of DRP	N (%)
Number of patients with DRPs	51 (33.6)
DRPs stratified by type	
Drug effectiveness	67 (84.8)
Adverse reactions	12 (15.2)
Drug cost	0 (0.0)
Unknown problem	0 (0.0)
DRP causes	
Drug selection	11 (14)
Drug form	0 (0.0)
Dose selection	57 (72.1)
Treatment duration	0 (0.0)
Drug use process	6 (7.6)
Logistics	4 (5)
Other	1 (1.3)
Intervention types	
No intervention	8 (10.1)
Intervention proposed and approved by the prescriber	64 (81)
Intervention proposed and not approved by the prescriber	2 (2.5)
Other: nurse	5 (6.3)

DRP - drug-related problem.

**Table 4:** Main drugs associated with the occurrence of drug-related problems

Type of DRP	Antimicrobials N (%)				
	Gentamicin	Cefazolin	Cefepime	Amikacin	Total
Patients with DRP	24 (55.8)	3 (7.0)	4 (9.3)	12 (27.9)	43 (100)
DRP stratified by type					
Drug effectiveness	20 (55.5)	2 (5.6)	4 (11.1)	10 (27.8)	36 (100)
Adverse reactions	4 (57.1)	1 (14.3)	0 (0.0)	2 (28.6)	7 (100)
Drug cost	0 (0.0)	0 (0.0)	0 (0.0)	0 (0.0)	0 (0.0)
Unknown problem	0 (0.0)	0 (0.0)	0 (0.0)	0 (0.0)	0 (0.0)
DRP causes*					
Drug selection	1 (50.0)	0 (0.0)	1 (50.0)	0 (0.0)	2 (100)
Dose selection	19 (52.7)	3 (8.4)	3 (8.4)	11 (30.5)	36 (100)
Drug use process	4 (80)	0 (0.0)	0 (0.0)	1 (20)	5 (100)
Intervention types					
No intervention	1 (50.0)	1 (50.0)	0 (0.0)	0 (0.0)	2 (100)
Intervention proposed and approved by the prescriber	19 (54.3)	2 (5.7)	3 (8.6)	11 (31.4)	35 (100)
Intervention proposed and not approved by the prescriber	2 (100.0)	0 (0.0)	0 (0.0)	0 (0.0)	2 (100)
Other: nurse	2 (50.0)	0 (0.0)	1 (25.0)	1 (25)	4 (100)

DRP - drug-related problem.

* DRP causes not shown because the frequency was zero: drug form, treatment duration, logistics and other.

Regarding the types of intervention, approximately 81% were classified as interventions proposed and approved by the prescriber, and only 2.5% were not approved. In addition to the prescriber, interventions were performed by the nursing team, and an incorrect dosage schedule and guidance regarding drug incompatibility were the main interventions related to this class.

## DISCUSSION

DRPs affected at least one third of the newborns receiving antimicrobial therapy in this study. The most vulnerable stage was the prescription step, with a predominance of DRPs related to drug effectiveness, with an emphasis on the involvement of aminoglycosides and cephalosporins.

The literature on the prevalence of DRPs in neonatology, specifically DRPs related to the use of antimicrobials, is scarce. However, the occurrence of prescription errors with this class of drugs ranges from 30 to 44% in this age group.^(13)^ In our study, the causes of DRPs were directly related to dose selection, indicating that the prescription step is the most vulnerable step. In fact, drug errors in intensive care generally predominate during the prescription step, especially inadequate doses and frequency of administration.^(14)^ Traditionally, the main classes involved with errors are antimicrobials, followed by antithrombotics, analgesics and agents acting on the renin-angiotensin system.^(7)^ The predominance of DRPs associated with effectiveness in our sample is mainly due to the prescription of doses below those recommended - something of concern, considering that a subdose of antimicrobials in neonatology implies a greater risk potential in these patients.^(15)^

In Brazil, Machado et al.^(16)^ evaluated prescribing errors in a NICU. The researchers observed that antimicrobials were the medications most frequently associated with inadequate prescriptions and that in addition to inadequate dosing, errors attributed to the use of diluents were also a significant problem.

During the therapeutic follow-up of the neonates, we observed significant variations in body weight over a few days, implying a constant need for dosage adjustment. This dosage complexity, attributed to pharmacotherapy in neonates, can be illustrated by aminoglycosides, antimicrobials that are widely used in neonatology.^(17)^ Gentamicin and amikacin were the main drugs associated with DRPs in our study; these DRPs mainly involved treatment effectiveness, especially subdoses and lack of compliance with the administration interval. In addition, DRPs classified as potential adverse reactions, such as the prescription of drugs in overdose, were also attributed to this class.

The peculiar pharmacokinetics of aminoglycosides, such as the increased distribution volume and renal elimination, require periodic adjustment of their dosage according to the neonate's development characteristics, such as changes in weight and gestational age.^(18)^

In this study, it was possible to observe that some neonates showed a 20% decrease or increase in body weight over a few days without an adjustment of the antimicrobial dosage. In addition, doses ten times below or above those recommended were also found. This is a common finding in other studies, since newborns are vulnerable to dose errors up to ten times more or less than desired due to the need for decimal dilution to adjust the volume to be administered.^(15)^

Similar to the situation for aminoglycosides, treatment with cephalosporins may also be compromised by inadequate dosage; however, these drugs are associated with a higher safety profile.^(19)^ Although cephalosporins are the second drug class most frequently associated with the occurrence of DRPs in this study, the occurrence was low.

In this sense, dose-related errors make the prescribing process difficult and affect the safety and efficacy of treatment in this group of patients specifically, since the use of low-dose antimicrobials is related to therapeutic failure and bacterial resistance.^(20,21)^

The occurrence of antimicrobial resistance, a worldwide problem that affects the increase in morbidity and mortality and the prolongation of hospitalization in the NICU, is associated with an inadequate use of antimicrobials.^(22)^ Shah et al.^(23)^ reported increased microbial resistance to aminoglycosides (gentamicin and amikacin) in the NICU. These drugs were identified in our study as the main ones implicated in DRPs associated with effectiveness and safety due to dose inadequacies. Therefore, we assume that rational use based on adequate drug selection and careful dosage schedules, with periodic monitoring by clinical pharmacists, is an important strategy in the fight against resistance.

In addition to dose inconsistencies, the lack of compliance with the interval between administrations was also a frequent finding, especially in relation to antimicrobials that require adjustment to be administered every 36 hours. Longer intervals may result in an insufficient minimal inhibitory concentration and favor therapeutic failure, as well as microbial resistance, as discussed above.^(24)^ In contrast, a decreased interval may lead to drug accumulation. This concern particularly affects this group of patients, in whom, for many reasons, drug clearance is impaired and, consequently, the half-life is prolonged, accentuating the increase in the drug serum concentration and exposing the patient to the risks of serious adverse reactions.^(21)^

Considering the DRPs classified as potential adverse reactions, which were also quite frequent in our sample, it is important to consider the pharmacokinetic characteristics of the drugs during the neonatal period. The functional immaturity of the newborn's organs leads to drug accumulation, since the drug elimination process has limited activity. Hepatic biotransformation is decreased,^(25)^ as well as glomerular filtration, considerably increasing the systemic exposure of the drugs.^(5)^ In addition, plasma protein binding may affect drug distribution and elimination, since in neonates, the concentration of proteins responsible for the transport of these compounds is lower than in adults. This difference contributes to higher drug concentrations remaining in the free fractions, increasing their distribution to tissues and contributing to increased toxicity.^(26)^ The greater amount of body water in these patients also plays a crucial role in neonatal pharmacokinetics. For example, hydrophilic drugs, such as gentamicin, have a higher distribution volume and a lower renal clearance rate; therefore, dosage adjustment should be judicious given the risk of rapid accumulation in the body and the occurrence of nephrotoxicity and ototoxicity - adverse reactions inherent in this drug class.^(24,26)^

Regarding the interventions performed, high approval by the prescriber was observed, demonstrating confidence in the work performed by the clinical pharmacist and how this professional can contribute positively to the reduction of DRPs. The benefit of the clinical pharmacist was confirmed in a systematic review that showed that pharmaceutical intervention can significantly reduce prescribing errors, as well as preventable adverse events in adult intensive care.^(27)^ Although the articles examined in that review were not focused on neonatology, we assume a similar potential for benefit in the NICU, since studies in neonatology are scarce.

The limitations of this study include the insufficient sample size for more accurate descriptive analysis and the fact that the study was conducted in a single location. It was also not possible to determine whether harm to the patient occurred, and all the analyses were based on potential DRPs. However, this study is relevant because of the scarcity of studies related to the subject in NICU in the literature, especially in Brazil. The prospective data collection and on-site evaluation contributed to the legitimacy of the variables analyzed.

## CONCLUSION

The drug-related problems associated with the use of antimicrobials in the studied neonates were classified mainly as problems related to drug effectiveness, particularly the prescription of drug subdoses, especially for aminoglycosides. We emphasize the importance of the clinical pharmacist as a key factor in the drug use process and the high approval of his/her interventions by the prescriber and other professionals.
